# Molecular insights into reproduction regulation of female Oriental River prawns *Macrobrachium nipponense* through comparative transcriptomic analysis

**DOI:** 10.1038/s41598-017-10439-2

**Published:** 2017-09-22

**Authors:** Hui Qiao, Hongtuo Fu, Yiwei Xiong, Sufei Jiang, Wenyi zhang, Shengming Sun, Shubo Jin, Yongsheng Gong, Yabing Wang, Dongyan Shan, Fei Li, Yan Wu

**Affiliations:** 1 Key Laboratory of Freshwater Fisheries and Germplasm Resources Utilization, Ministry of Agriculture, Freshwater Fisheries Research Center, Chinese Academy of Fishery Sciences, Wuxi, 214081 China; 2 Wuxi Fishery College Nanjing Agricultural University, Wuxi, 214081 China; 3 College of Fisheries and Life Sciences, Shanghai Ocean University, Shanghai, 201306 China

## Abstract

The oriental river prawn, *Macrobrachium nipponense*, is an important commercial aquaculture resource in China. During breeding season, short ovary maturation cycles of female prawns cause multi-generation reunions in ponds and affect the growth of females representing individual miniaturization (known as autumn -propagation). These reproductive characteristics pose problems for in large - scale farming. To date, the molecular mechanisms of reproduction regulation of *M*. *nipponense* remain unclear. To address this issue, we performed transcriptome sequencing and gene expression analyses of eyestalk and cerebral ganglia of female *M. nipponense* during breeding and non-breeding seasons. Differentially expressed gene enrichment analysis results revealed several important reproduction related terms and signaling pathways, such as “photoreceptor activity”, “structural constituent of cuticle” and “G-protein coupled receptor activity”. The following six key genes from the transcriptome were predicted to mediate environmental factors regulating reproduction of *M. nipponense*: neuroparsin, neuropeptide F II, orcokinin II, crustacean cardioactive peptide, pigment-dispersing hormone 3 and tachykinin. These results will contribute to a better understanding of the molecular mechanisms of reproduction of oriental river prawns. Further detailed functional analyses of the candidate reproduction regulation related neuropeptides are needed to shed light on the mechanisms of reproduction of crustacean.

## Introduction

**Figure 1:**
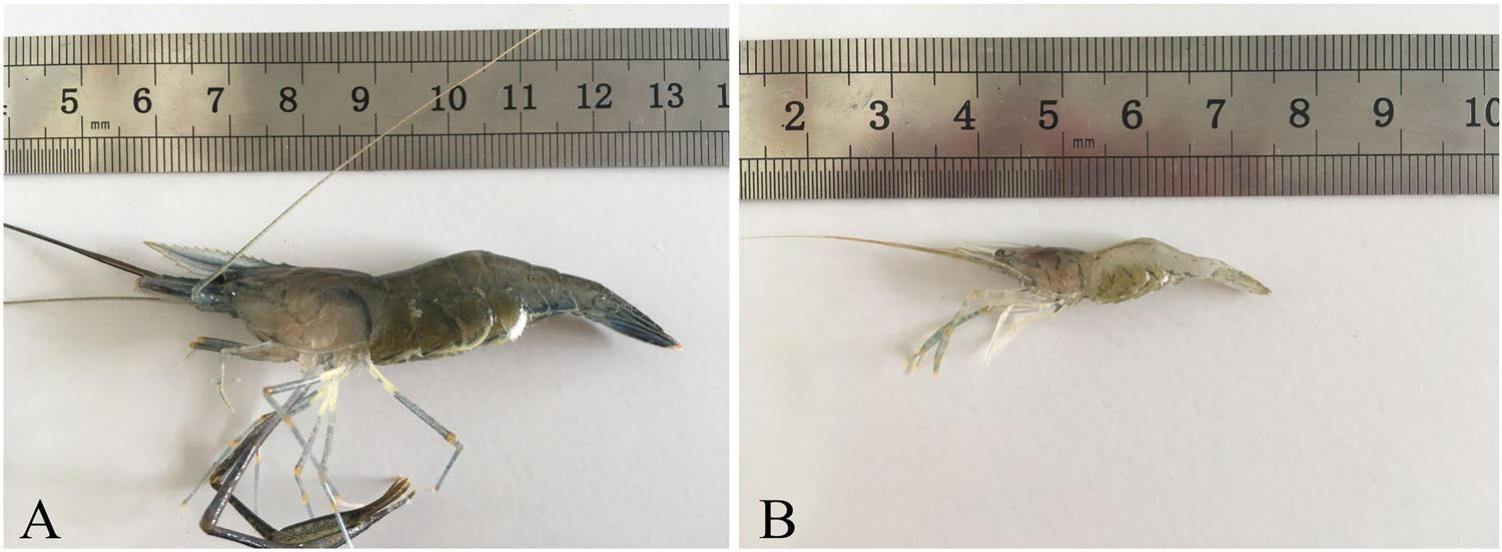
Normal and autumn-propagation female individuals of *M. nipponense* with eggs. Notes: (**A**) is a normal female prawn of *M. nipponense* with eggs and (**B**) is an autumn-propagation female prawn of *M. nipponense* with eggs.

The oriental river prawn *Macrobrachium nipponense* (Decapoda, Palaemonidae), is widely distributed in Japan, Korea, Vietnam and Myanmar^1,2^. It is also an important commercial fishery resource for aquaculture in China with an annual cultured production of about 265,061 tons and an annual cultured output value of near 20 billion RMB in 2015^3,4^. Prawn aquaculture in China has entered into a rapid development period and become a very important sector in Chinese agriculture. However, the reproductive characteristics of *M. nipponense* pose problems for large scale farming. During breeding season, especially in late April, female oriental river prawns enter into period of a rapid development, displaying a short ovary maturation cycle and embryo and larval development are accelerated as water temperature increases. In August -October, the newborn females can mate and lay eggs even though they hatched only 45 days and are no more than 3 cm long. This sexual precocity phenomenon, also known as autumn -propagation, is very prevalent in both wild and farmed prawns (Fig. 1). It causes multi-generation reunions in ponds, which block pure line construction. It also increases breeding density, which can affect oxygen concentration and lead to greater feed consumption, thereby impacting breeding. Autumn propagation also affects the growth of female prawns, resulting in individual miniaturization and subsequent low market value of the product^5^.

The mechanisms that regulate reproduction in crustaceans have become a hot research topic in recent years. Advances in understanding insulin-like peptides in crustaceans and the ability to manipulate them and their encoding transcripts (IAGs) have raised the possibility of sexually manipulating crustacean populations^6^. However, our understanding of the molecular regulatory mechanisms involved in crustacean reproduction is still far from complete. Eyestalk and cerebral ganglia are known to be major sites for production and secretion of many hormones involved in crustacean reproduction^7,8^. Previous studies showed that the X-organ-sinus gland complex system in the eyestalk and cerebral ganglia secretes many important neuropeptide hormones (e.g., crustacean hyperglycaemic hormone, molt inhibiting hormone, vitellogenesis inhibiting hormone and mandibular organ-inhibiting hormone), pigment regulatory peptides (e.g., pigment-dispersing hormone, red pigment-concentrating hormone) and neurotransmitters (e.g., serotonin, 5-HT and dopamine)^9–11^. In addition, transcriptome sequencing has led to the discovery of important reproduction-related genes^12–17^. As a consequence, fairly long lists of neuropeptides are available for several decapods^18–20^. Large-scale next-generation sequencing studies of mixed tissues (including the eyestalk), ovary, testis, and the androgenic gland from *M. nipponense* have been conducted to identify reproduction- and ovary development-related genes^21–24^, such as vitellogenin^25^, insulin-like androgenic gland hormone-binding protein^26^, sperm gelatinase^27^, gonadotropin-releasing hormone like receptor^28^ and heat shock proteins^29^. However, the transcriptomic data that come from independent eyestalk and cerebral ganglia of *M. nipponense* was seldom reported.

In this paper, we present the comparative transcriptomes of eyestalk and cerebral ganglia from female *M. nipponense* in breeding and non-breeding season. The objectives of this study were to: (1) construct eyestalk and cerebral ganglia transcriptomes of female *M. nipponense* during breeding and non-breeding seasons; (2) identify differentially expressed genes (DEGs) and pathways that may play important roles in sexual precocity in *M. nipponense*; and (3) gain insight into the regulatory mechanisms involved in crustacean reproduction. The results of this work will contribute to a better understanding of the molecular mechanisms of reproduction of oriental river prawns.

## Methods

### Prawn material

Healthy adult female oriental river prawns of one-year-old, each approximately 2.26 ± 0.59 g in wet weight, were obtained from Freshwater Fisheries Research Center, Chinese Academy of Fishery Sciences, Wuxi, China.

Approximately 50 non-breeding season female prawns were captured in January, 2015 and cultured in a re-circulating water aquarium system and fed with paludina twice per day at water temperature (10 °C) for 3 days before sample collection. Prawns were anesthetized under MS222 anesthesia to minimize suffering prior to dissections. Five prawns formed one biological replicate, and three biological replicates were performed for each sampling time. Fresh cerebral ganglia and eyestalk tissue from each prawn, named as non-breeding season cerebral ganglia and eyestalk (NBSB and NBSE), was quickly collected and immediately and stored at −80 °C until processed.

About 100 breeding season individuals with different ovarian stages were obtained in June, 2015 and maintained under the same condition at water temperature (24 °C) for 3 days before sample collection. The ovarian cycle of prawns was classified into five stages based on the previous results^30^ and ovary color observation: Stage I (undeveloped stage, oogonium proliferation, transparent), Stage II (developing stage, primary vitellogenesis, yellow), Stage III (nearly-ripe stage, secondary vitellogenesis, light green), Stage IV (ripe stage, vitellogenesis termination, dark green) and Stage V (spent stage, gray). Prawns were anesthetized under MS222 anesthesia to minimize suffering prior to dissections. One prawn each of five ovarian stages formed one biological replicate, and three biological replicates were performed for each sampling time. Fresh cerebral ganglia and eyestalk tissue from each prawn, named as breeding season cerebral ganglia and eyestalk (BSB and BSE), was quickly collected and immediately and stored at −80 °C until processed. For qRT-PCR validation, five individual prawns were sampled and pooled into one at each ovarian stage and fresh cerebral ganglia and eyestalk tissue from each prawn was quickly collected and immediately stored at −80 °C for RNA isolation. Our study does not involve endangered or protected species. This study was approved by the Institutional Animal Care and Use Ethics Committee of the Freshwater Fisheries Research Center, Chinese Academy of Fishery Sciences (Wuxi, China).

### RNA extraction, sequencing library preparation and next-generation sequencing

Total RNA from eyestalks and brains of non-breeding and breeding season was isolated using RNAiso Plus Reagent (TaKaRa, Japan) according to the manufacturer’s protocols. The RNA was then treated with RNase free DNase I (Takara, Japan) to avoid DNA contamination. The RNA integrity and quantity were determined using an Agilent 2100 Bioanalyzer (Agilent, Shanghai, China) and a NanoDrop 1000 spectrophotometer (Thermo Scientific, USA).

Poly (A) mRNA was briefly isolated using oligo (dT) beads. Short mRNA fragments were used as templates to synthesize the first-strand cDNA with random hexamers. The second-strand cDNA was synthesized using buffer, dNTPs, RNase H and DNA polymerase I. Short fragments were purified with Takara’s PCR extraction kit (Takara, Japan). Sequencing adapters were ligated to short fragments and resolved by agarose gel electrophoresis. Proper fragments were selected and purified and subsequently PCR amplified to create the final cDNA library template. A mixed cDNA sample representing three individuals s of tissues was prepared and sequenced using an Illumina HiSeq™ 2500 with 150 bp pair-end reads produced.

### *De novo* assembly and annotation functional of the transcriptome

All the raw reads were initially processed to obtain clean reads using the following three steps. (a) Reads with adaptor contamination were discarded; (b) reads with ambiguous sequences “N” larger than 5% were removed; (c) low quality reads that contained more than 20% *Q* < 20 bases were discarded^31^.

The clean reads were assembled into non-redundant transcripts using the Trinity, which has been developed specifically for the de novo assembly of transcriptomes using short reads^32^. First, clean reads with a certain length of overlap were combined to generate contigs. If there were multiple duplicated reads, the redundant duplicated reads were removed and only one read was retained for assembly. Then, the paired-end reads were realigned to contigs to obtain transcripts, which could identify different contigs in the same transcript and ensure the interval among these contigs. The TGICL program (http://compbio.dfci.harvard.edu/tgi/software/) was then used to delete redundant transcripts and further assemble all transcripts to form a single set of unigenes. Finally, all of the clean reads were pooled together and assembled to form the global transcriptome data of *M. nipponense*.

For annotation, the assembled unigenes were annotated by the public protein databases, including, Swissprot protein (http://www.expasy.ch/sprot), clusters of orthologous groups (COG; http://www.ncbi.nlm.nih.gov/), non-redundant (NR; http://www.ncbi.nlm.nih.gov) and the Kyoto encyclopedia of genes and genomes (KEGG; http://www.genome.jp/kegg) databases using BLAST (E-value < 10^−5^). The Getorf software was used to predict coding regions of the assembled unigenes^33^. Based on Nr annotation, BLAST2 GO program was used for GO analysis (http://www.geneontology.org/). Further, COG classification and signal pathway annotation of unigenes was performed by BLASTx searching against the COG database and KEGG database.

### Differential expression analysis

The expression of all unigenes was estimated by calculating read density as ‘reads per kb per million reads’ (RPKM) using the RSEM program^34^. To identify the DEGs, FDR ≤ 0.001 and two-fold change (log2 Ratio) ≥ 1 or ≤−1 were set to be the threshold for judging the significance of gene expression differences^35^. The gene expression profiles were compared with each other, and then all DEGs in each comparison were carried on the GO functional and KEGG pathway enrichment analysis using GO database and KEGG database^36^. The DEGs mapped to the KEGG database were screened and analyzed by MapMan software.

### The identification of the simple sequence repeats (SSRs) and single nucleotide polymorphism markers (SNPs)

Microsatellite identification software (http://pgrc.ipk-gatersleben.de/misa/) was used to identify SSR markers. The repeat thresholds for mono-, di-, tri-, tetra-, penta- and hexa- nucleotide motifs were set as 10, 6, 5, 5, 5 and 5, respectively. The maximum number of bases interrupting two SSRs in a compound microsatellite was set as 100. The identification of SNPs for each library used SOAPsnp (http://soap.genomics.org.cn/soapsnp.html)^37^.

### Real-time quantitative RT-PCR verification

The DEGs results were validated using qRT-PCR. The qRT-PCR assays were performed with three replicates, and the actin gene was used as an internal control to normalize the expression level of the target genes. The specific primers were designed according to the unigene sequences using Primer 5.0 software (Table S1).

Amplifications were performed on a 96-well plate with a 25 μL reaction volume containing 1 μL cDNA (50ng), 10 μL SsoFastTM EvaGreen® Supermix (BIO-RAD, CA, USA), 0.5 μL 10 μM of primers, and 13 μL of ddH_2_O. The PCR temperature profile was 95 °C for 30 s followed by 40 cycles of 94 °C for 15 s, 58 °C for 20 s and 72 °C for 20 s, with a 0.5 °C/5 s incremental increase from 60 °C to 95 °C. The relative copy number of mRNA was calculated according to the 2^− ∆∆CT^ comparative CT method^38^. Quantitative data were expressed as means ± SD. Two-side t test was used to compare expression levels. A probability level of 0.05 was used to indicate significance. All statistics were performed using SPSS Statistics 19.0.

## Results

### Sequencing and *de novo* assembly

A total of 300,308,758 (about 89.85 Gb) clean reads representing 89,847,943,292 clean nucleotides (nt) were produced. Each sample yielded more than 7 Gb of data (at least 20 million reads). The average Q30 percentage and GC content were 91.03% and 46.49%, respectively (Table 1).

**Table 1:** Statistics of sequencing data across the 12 libraries in *M. nipponense*.

Sample ID	Clean Reads^a^	Clean Data^b^	GC content (%)	Q30 ratio (%)^c^
BSE-1	35,472,264	10,641,679,200	47.95	92.87
BSE-2	27,726,160	8,289,681,005	48.16	91.51
BSE-3	25,885,516	7,739,075,785	48.18	90.86
BSB-1	22,221,188	6,666,356,400	46.83	92.19
BSB-2	20,949,606	6,253,112,661	47.17	90.31
BSB-3	20,135,071	6,005,937,911	47.15	90.59
NBSE-1	30,205,549	9,061,664,700	44.64	91.77
NBSE-2	26,539,323	7,926,033,602	44.93	90.33
NBSE-3	25,168,207	7,515,926,673	44.96	89.65
NBSB-1	23,805,615	7,141,684,500	45.85	91.95
NBSB-2	21,591,224	6,449,914,216	46.04	90.51
NBSB-3	20,609,035	6,156,876,639	46.07	89.83
Sum	300,308,758	89,847,943,292	/	/
Average	25,025,729	7,487,328,608	46.49	91.03

Notes: BSE-1-BSE-3 and BSB-1- BSB-3 represent three replications for prawn eyestalks and brains of breeding season; NBSE-1-NBSE-3 and NBSB-1- NBSB-3 represent three replications for prawn eyestalks and brains of non-breeding season.

^a^Clean Reads: The number of paired-end Reads in Clean Data.

^b^Clean Data: The total number of the bases in Clean Data.

^c^Q30 ratio (%): The percentage of the base whose Clean Data quality value is at least 30.

De novo sequence assembly generated 118,366 transcripts with a total length over 120,087,867 bp. The mean transcript size was 1,014.55 bp, and the transcript N50 was 1,698 bp. Finally, 90,491 unigenes were obtained after combining the transcripts, with a total length of 73,722,132 bp. The mean unigene length was 814.69 bp, and the N50 was 1,091 bp. In these unigenes, 52.68% were 300–500 bp, 27.60% were 500–1,000 bp, 11.96% were 1,000–2,000 bp, and 7.76% were >2,000 bp in size. An overview of the assembly results is provided in Tables 2 and 3. The raw data were uploaded to the National Center for Biotechnology Information (NCBI, PRJNA339889).

**Table 2:** Length distribution of the transcripts and unigenes from the de novo assembly.

Length range (bp)	Transcripts	Unigenes
300–500	54,871 (46.357%)	47,670 (52.679%)
500–1000	31,468 (26.585%)	24,977(27.602%)
1000–2000	17,201 (14.53%)	10,822 (11.959%)
>2000	14,826 (12.526%)	7,022 (7.760%)

**Table 3:** Assembly statistics for the transcripts and unigenes.

	Transcripts	Unigenes
Total number	118,366	90,491
Total length	120,087,867	73,722,132
N50 length	1698	1091
Mean length	1014.55	814.69

Notes: N50 indicates the length of the smallest transcripts in the set that contain the fewest (largest).

### Functional annotation

In total, 34,183 unigenes significantly matched a sequence at a cut-off E-value of 10^−5^ in at least one of the public databases, which included Cluster of Orthologous Groups (COG), Gene Ontology (GO), Kyoto Encyclopedia of Genes and Genomes (KEGG), Swissprot and NCBI non-redundant (NR). Of these, 22,282 unigenes over 300 bp were annotated and 11,901 unigenes larger than 1000 bp could be matched to known proteins. In total 33,012 unigenes matched NCBI NR annotations, which contained more annotated genes than the other databases (Table S2). Species most represented in the BLASTx searches included *Zootermopsis nevadensis* (2858), *Daphnia pulex* (1638) and *Stegodyphus mimosarum* (1033), but “other” species was the largest group (22279) (Fig. S1).

To analyze the functions of these unigenes, GO assignments were made. 13,295 unigenes were assigned at least one GO term, and all GO terms were classified into three groups and further divided into 55 functional subgroups. Among biological processes, more transcripts were assigned to “metabolic process” (7,742) and “cellular process” (6,478) than to other terms. Among the “molecular functions”, “binding-related genes” (6,339) and “catalytic activity” (6,278) were the most enriched GO terms. Among “cellular components”, transcripts assigned to “cell part” (4,545), “cell” (4,507) and “organelle” (3,086) were the three most abundant (Fig. S2).

When the assembled unigenes were annotated by the COG database, 9,646 unigenes with significant homology were classified into 25 COG clusters. Among the functional clusters, “general function prediction only” was the largest cluster (2,705 unigenes) and “extracellular structures” (1unigene) was the smallest one (Fig. S3).

To analyze the involved signal pathways, the unigenes were annotated by the KEGG database. A total of 9,243 unigenes were assigned to 221 KEGG pathways. The largest pathway group was protein processing in the “Ribosome” (ko03010), which contained 832 genes. This was followed by protein processing in “endoplasmic reticulum” (ko04141) and “RNA transport” (ko03013), which contained 340 and 280 genes, respectively.

### Development of simple sequence repeats (SSR) and single nucleotide polymorphism (SNP) markers

In total, 14,899 SSRs were identified. They were located in 17,844 unigenes greater than 1 Kb in size, and 3667 unigenes contained more than one SSR (Table S3). The largest fraction of SSRs identified consisted of mono-nucleotides (7953), followed by di-nucleotides (3382) and tri-nucleotides (3357).

We also detected SNPs in each library (Table S4). In breeding season group, we found that the number of heterozygous SNPs (Hete SNPs) was larger than the homozygous SNPs (Homo SNPs) in eyestalk transcriptomes, whereas the opposite was true in brain transcriptomes. However, in non-breeding season group, the number of Hete SNPs was much lower than that of the Homo SNP in both eyestalk and cerebral ganglia transcriptomes.

### Global analysis of DEGs

**Figure 2:**
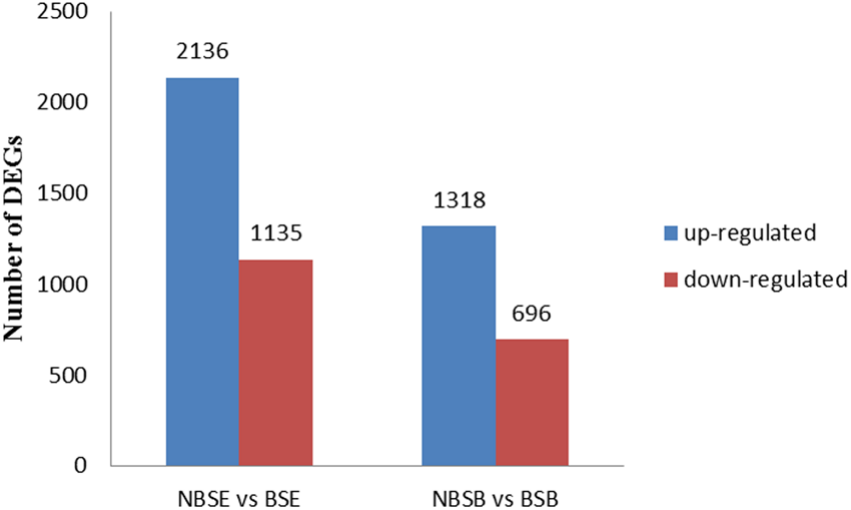
Numbers of DEGs DEGs in NBSE vs. BSE and NBSB vs. BSB. Notes: Up- and down-regulated unigenes are shown in blue and red, respectively. The x-axis shows two comparisons. The y-axis represents the total number of DEGs.

We calculated and compared expression levels of DEGs among the four groups to identify reproduction-related genes: cerebral ganglia of breeding season (BSB), eyestalks in breeding season (BSE), cerebral ganglia of non-breeding season (NBSB) and eyestalks of non-breeding season (NBSE). All DEGs with the absolute value of log2 Ratio ≥1 and the false discovery rate (FDR ≤ 0.001) are listed in Table S5. A total of 3,271 DEGs were detected for the NBSE and BSE, of which 2,136 and 1,135 unigenes were up-regulated and down-regulated respectively. For the NBSB and BSB comparison, 2,014 DEGs were detected, of which 1,318 and 696 unigenes were up-regulated and down-regulated respectively (Fig. 2).

**Figure 3:**
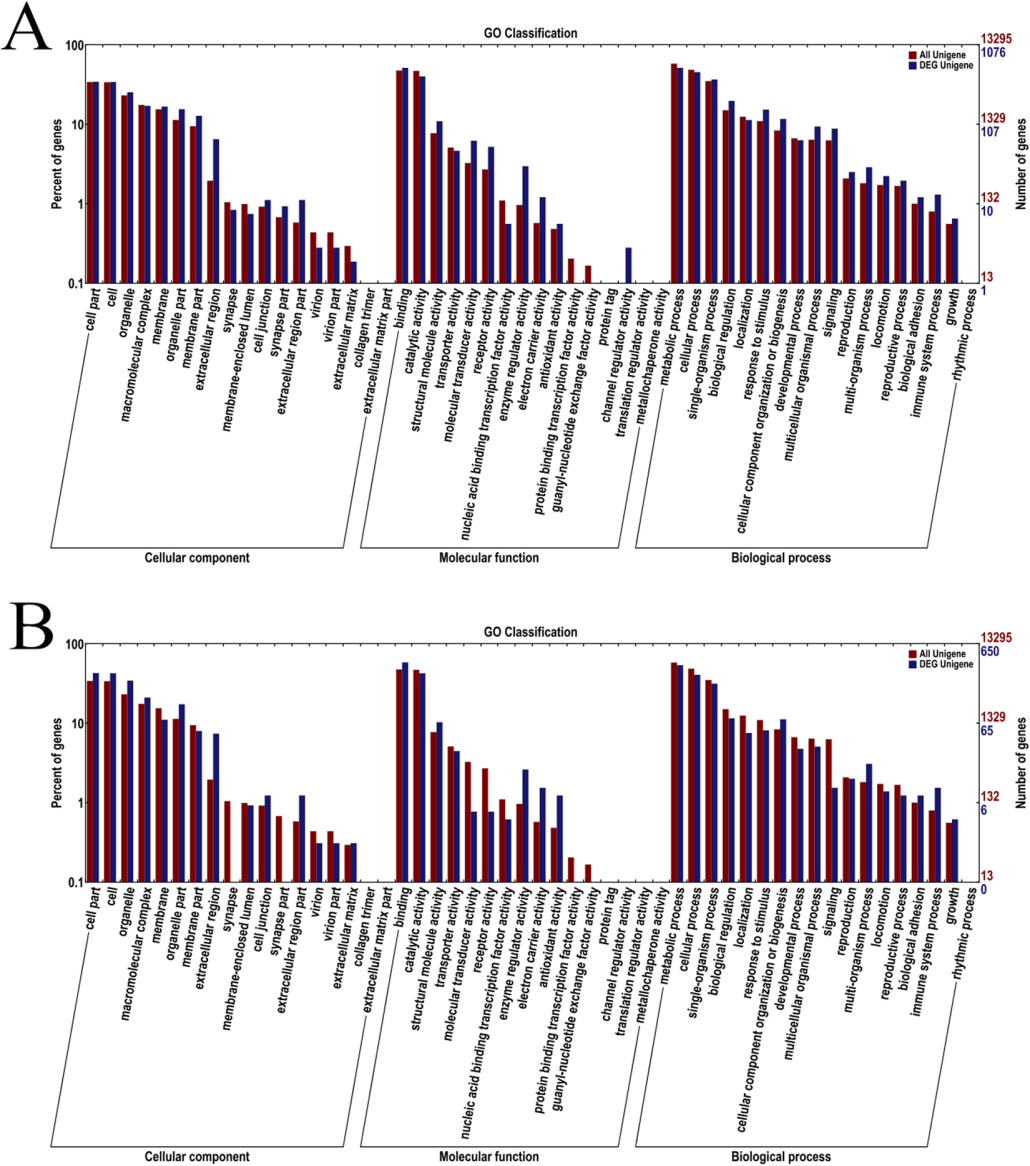
The GO classification analyses of DEGs in NBSE vs. BSE and NBSB vs. BSB. Notes: A is NBSE vs. BSE and B is NBSB vs. BSB.

Functional annotation of DEGs at a cut-off E-value of 10–5 was performed using COG, GO, KEGG, Swissprot and NCBI NR databases. Table S6 list the statistical results. GO functional annotation was performed to identify the biological function of DEGs (Fig. 3). Fewer of DEG unigenes had cellular component functions compared with the total group of unigenes. In addition, the annotations of functions of DEG unigene and all unigene for NBSE vs. BSE and NBSB vs. BSB comparisons were consistent with each other.

GO enrichment analysis was performed to identify significantly over-represented GO terms in the DEGs with a *p*-value < 0.05 and FDR < 0.01. The top 10 significant GO terms listed in Table S7. These terms included “photoreceptor activity”, “structural constituent of cuticle”, “G-protein coupled receptor activity”, “structural constituent of cytoskeleton”, “protein phosphatase type 2A regulator activity”, “iron ion binding”, “calcium ion binding”, “peptidase inhibitor activity”, “pigment binding” and “GTP binding”.

**Figure 4:**
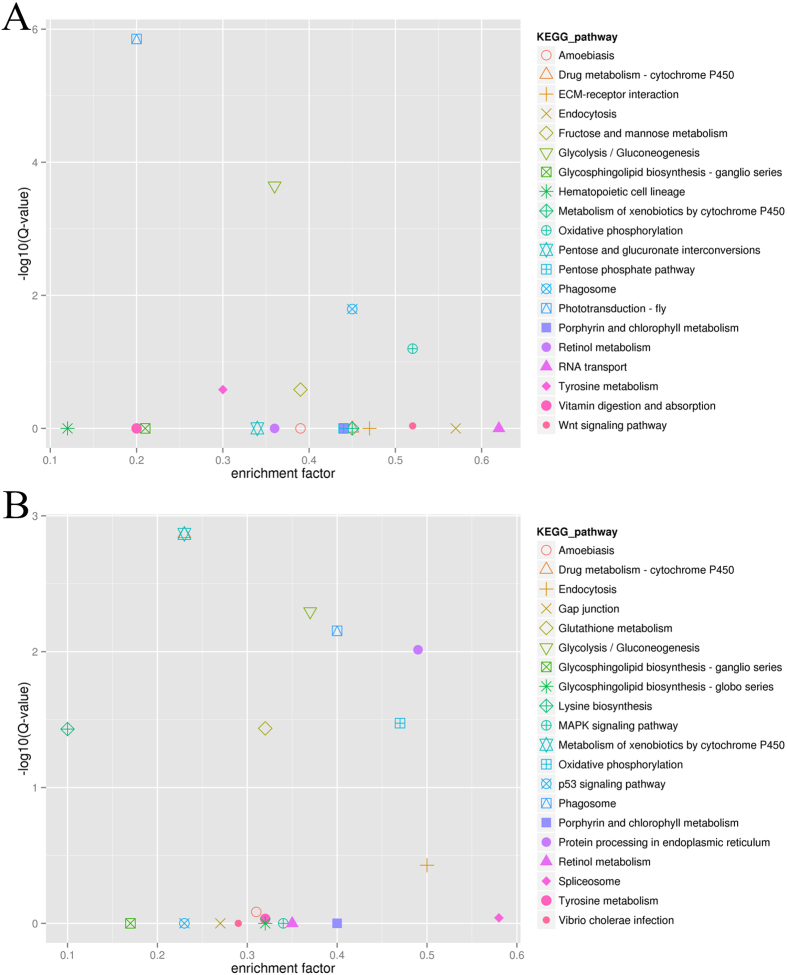
Statistics of KEGG pathway enrichment of DEGs in NBSE vs. BSE and NBSB vs. BSB. Notes: The abscissa value represented rich factor which means DEG/unigene of pathway term; the ordinate value represented –log 10 (Q-value). (**A**) is NBSE vs. BSE and (**B**) is NBSB vs. BSB.

**Figure 5:**
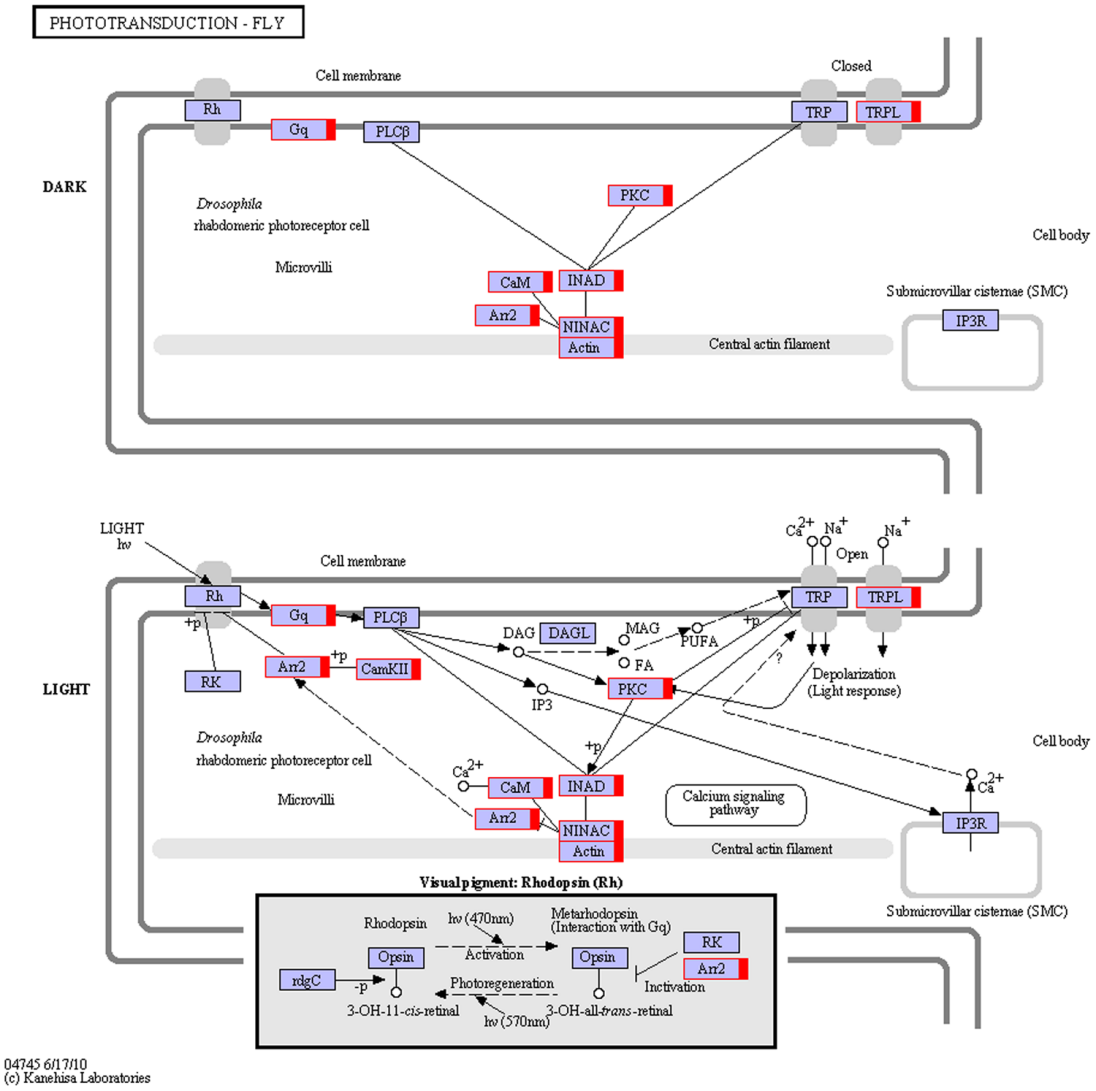
Significantly differentiated expressed genes that were identified by KEGG as involved in the “Phototransduction – fly” signaling pathway in NBSE vs. BSE. Notes: Red boxes indicate significantly increased expression. Black boxes indicate unchanged expression. Based on KEGG map04745^36^ (http://www.genome.jp/kegg-bin/show_pathway?ko04745).

To better understand the reproduction- related pathways, DEGs also were analyzed using the KEGG database for pathway enrichment. We evaluated 20 most significant enriched pathways with the *Q*-value < 0.05 for the NBSE vs. BSE and NBSB vs. BSB comparisons (Fig. 4). For the eyestalk, the significantly enriched pathways between breeding season and non-breeding season included “phototransduction-fly” (Fig. 5), “glycolysis/gluconeogenesis” and “phagosome”. The most significantly enriched pathways for the cerebral ganglia between breeding season and non-breeding season were “drug metabolism– cytochrome P450” and “metabolism of xenobiotics by cytochrome P450”. Many other pathways, including “glycolysis/gluconeogenesis”, “phagosome”, “protein processing in endoplasmic reticulum”, “oxidative phosphorylation”, “glutathione metabolism” and “lysine biosynthesis”, were also enriched. Interestingly, when comparing eyestalk and cerebral ganglia of non-breeding season vs. breeding season, the DEGs were both concentrated most significantly in “phototransduction – fly”.

### Neuropeptides discovery by transcriptome mining and differentially expressed analyses

Further transcript characterization was performed for selected neuropeptides that were thought to play an important role in crustacean molting, feeding, metabolism, growth, and reproduction. Twenty-two transcripts encoding neuropeptide precursors were identified from the transcriptome data (Table S8).

The deduced peptides include red pigment concentrating hormone (RPCH), crustacean hyperglycemic hormone (CHH), molt inhibiting hormone (MIH), gonad inhibiting hormone (GIH), isoforms of allatostatin-B- I(AST-B-I), allatostatin B II I(AST-B- II), allatostatin-C(AST-C), neuropeptide F I(NPF-I), neuropeptide F II(NPF-II), short neuropeptide F (sNPF), sulfakinin, neuroparsin (NP), orcokinin II, crustacean cardioactive peptide (CCAP), eclosion hormone 2 (EH2), pigment dispersing hormone I (PDH-I), pigment-dispersing hormone 3(PDH3), tachykinin(TK), calcitonin-like diuretic hormone, corazonin preprohormone, crustacean female sex hormone and FLRFamide precursor protein B. Among these peptides, NP was found to be significantly up-regulated in the NBSE vs. BSE and down-regulated in the NBSB vs. BSB comparisons. NPF-II, orcokinin II, CCAP, PDH3 and TK were significantly down-regulated in the NBSE vs. BSE comparison. Sulfakinin were only various in the NBSE vs. BSE comparison.

### Experimental validation

**Figure 6:**
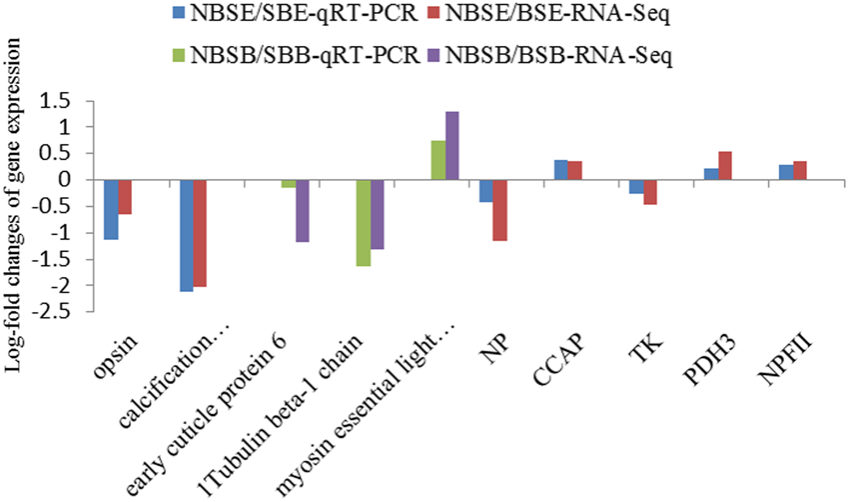
Comparison of gene expression patterns obtained using RNA-Seq and qRT-PCR. Notes: Log-fold changes are expressed as the ratio of gene expression after normalization to β -actin.

To validate the veracity and reliability of DEGs identified by RNA-Seq, we further examined relative expression levels of 10 genes of interest and quantified their relative expression folds between NBSE, BSE, NBSB and BSB groups by RT-qPCR. A total of 20 prawns from the NBSE, BSE, NBSB and BSB groups (n = 5 in each group) were used for each gene validation. The expression analysis was performed for selected genes belonging to the phototransduction, structural constituent of cuticle, and cytoskeleton biosynthesis pathways, including early cuticle protein 6, opsin, and calcification associated soluble matrix protein 2, tubulin beta-1 chain and myosin essential light chain. The qRT-PCR expression patterns of the 10 selected DEGs were in agreement with the results of RNA-Seq analysis (Fig. S4). In addition, a few transcripts encoding NP, CCAP, TK, PDH3 and NPFII showing differential expression, were also selected randomly from the transcriptome dataset and validated via real-time PCR analysis. A comparative analysis of all selected genes revealed similar expression pattern for real-time qPCR and RNA-seq data, illustrating the consistency of the results (Fig. 6).

## Discussion

### Transcriptomes of female *M. nipponense* eyestalk, and cerebral ganglia

Previous transcriptome analyses provide an insight into sex and reproduction of *M. nipponense* ^23,24^. In order to produce a comprehensive reference transcriptome, this study established a repertoire of annotated genes and DEGs from eyestalks and cerebral ganglia of female oriental river prawns during breeding and non-breeding seasons. From the four groups, 90,491 unigenes were assembled and investigated. Only 37.75% (34,183) of the unigenes significantly match with other known genes in the reference databases. BLAST results of the NR database showed that the majority of *M. nipponense* unigenes had the highest number of matches with the insect *Z. nevadensis* followed by the crustacean *D. pulex*. This result was similar to that reported for the *M. nipponense* ovary transcriptome but different from that reported for the *M. rosenbergii* eyestalk, CNS, and ovary transcriptome^8,29^. GO functional annotation and KEGG analysis revealed a great diversity of genes involved in various molecular functions.

### Major biological associations explored by enrichment analysis

#### Photoreceptors

topGO term and pathway enrichment analyses were performed to explore GO terms and pathways in the DEGs related to ovarian maturation regulation. Comparison of proportions of GO and KEGG functional descriptions indicated that the enrichment trend was differed significantly between the DEGs and all genes. Both methods identified the same important signaling pathways: “photoreceptor activity” for GO term enrichment and “Phototransduction-fly” for KEGG enrichment. Light is primarily a source of energy for organisms, and it also can be acted as an environmental signal to control metabolic and reproductive processes^39^. In both birds and mammals, the photoperiodic pathways can regulate thyrotrophin secretion, which in turn regulates gonadotropin-releasing hormone secretion^40,41^. In invertebrates, the role of photoreceptors in insects also plays key roles in mates and other specific behaviors^42^. In this transcriptome data base, more than 300 elements belonging to the rhodopsin family were detected, and most of them were the part of DEGs designated as “photoreceptor activity” and “phototransduction-fly” term (opsin and amelanopsin). Similar results were also reported for the transcriptome of the crayfish *Procambarus clarkii* ^43^. In crustaceans, previous studies showed that the photoperiod has a strong induction effect on the gonad development^44,45^. However, no report about how light regulate reproduction are available. Based on results of this study, we speculate that a photoperiodic pathway may regulate in the reproduction crustaceans, as is true for birds and mammals. However, this hypothesis will require further studies to clarify the function of these rhodopsin family genes and elucidate the relationship between these photoreceptors and reproduction.

#### Cuticle proteins

Other inportant GO terms identified in this study are “structural constituent of cuticle” and “G-protein coupled receptor activity”. More than 110 cuticle related genes are included in the “structural constituent of cuticle” term including cuticle protein, early cuticle protein, strongly chitin-binding protein-1, and structural constituent of cuticle and calcified cuticle protein. Cuticular proteins are essential elements in cuticle occurrence and differentiation^46,47^. They play an indispensable role in insect cuticle integration, body shaping and other physiological process^48–50^. *M. nipponense* females cannot lay eggs unless they slough their old skin during breeding seasons which suggests a link between molting and reproduction. Molting is an important event for arthropod and ecdysteroids are multifunctional hormones in male and female arthropods regulate the construction of important parts and organs of insect body^51^. Previous studies have shown that ecdysteroid biosynthesis and its hormonal regulation are demanded for insect gonads^52^. Thus,Much needed be done to investigate the correlation between molting and reproduction in *M. nipponense* requires further study.

### Reproduction regulation related neuropeptides identified by transcriptome mining

With the advancement of technology, such as genome and transcriptome mining and mass spectrometry, more than two dozen neuropeptide families have been characterized from various crustacean species^53^. Detail information about these peptides and their potential reproductive function of these neuropeptides is available for some insects^54–58^. In the current study, 22 transcripts encoding neuropeptide precursors were identified from the transcriptome data. However, only six neuropeptides (NP, CCAP, PDH, NPF–II, orcokinin–II, and TK) exhibited significant differences in the NBSE vs. BSE and NBSB vs. BSB comparisons. The other neuropeptides seemed not to react very strongly to seasonal alternation.

In this study, NP levels differed significantly varied in both the NBSE vs. BSE and NBSB vs. BSB comparisons. NPs, which had several isoforms, have been proved to play an important role in regulating reproduction of insects and crustaceans. They were first characterized from the brain of the locust, they were found to exhibit a vitellogenesis-inhibiting effect, preventing oocyte growth^59–61^. In crustaceans, the only exception of NP in *Metapenaeus ensis* that the gene silencing caused a significant decrease of vitellogenin expression level in both hepatopancreas and ovary^62^. Our results provide further evidence that NP might be involved in seasonal reproductive regulation. The main difference lies between breeding and non-breeding season was the variation of environmental factors. Light, temperature, and nutrition may be the most important factors affecting animal reproduction. Based on our results, we hypothesized that neuroparsin might mediate the way in which environmental factors regulating reproduction of *M. nipponense*.

Expression levels of the other five neuropeptides differed significantly between breeding season and non-breeding season neuropeptides only in the eyestalk. CCAP, which is found in neurons within the central nervous system of crustaceans and insects, was reported to play a role in accelerating heart contractions and hemolymph velocity^58^. It is also involved in modulating oviduct contraction in *L. migratoria* ^63^ and was significantly up-regulated in the late vitellogenic stage in *S. paramamosain* ^64^. PDH and RPCH are light adapting hormones that control circadian rhythm^65,66^. Several studies reported that PDH expression was maintained at high levels in the cerebral ganglia in the early vitellogenic stage but decreased significantly in the late vitellogenic stage in *S. paramamosain* ^64,65^. Neuropeptide F II (NPF-II), orcokinin II, and tachykinin (TK) have recently been reported by transcriptomes. In the cerebral ganglia transcriptome of *S. paramamosain* ^66^, differential expression levels of NPF-II, orcokinin II, and TK in the cerebral ganglia at different vitellogenic stages indicated that they may play a role in crustacean reproduction. However, the reproductive al function of these neuropeptides remains unknown and warrants further research. In the current study, significant expression differences of these neuropeptides were found only in the eyestalk, and phototransduction was the most important pathway. Based on this finding, we hypothesize that light might have a dominant effect on eyestalk neuropeptides secretion and that these five neuropeptides might mediate light regulating reproduction of *M. nipponense*. We currently are conducing further studies in our lab to test these hypotheses.

In summary, the comparative transcriptomes of eyestalk and cerebral ganglia from female *M. nipponense* during breeding and non-breeding seasons were evaluated in this study. We predicted major biological terms related to reproduction, such as photoreceptor activity and cuticle proteins, based on enrichment analysis. We also identified a series of peptides, including NP, CCAP, PDH, orcokinin II, TK, and NPF-II, which might mediate environmental factors that regulate reproduction of *M. nipponense*. These results provide insights into the mechanisms involved in reproduction of *M. nipponense*.

## Electronic supplementary material

Supplementary Info
