# Supplementary material for: Molecular insights into reproduction regulation of female Oriental River prawns Macrobrachium nipponense through comparative transcriptomic analysis
Source: Sci Rep. 2017 Sep 22;7:12161. doi: 10.1038/s41598-017-10439-2 (PMC5610250; doi:10.1038/s41598-017-10439-2)

Molecular insights into reproduction regulation of female Oriental River prawns *Macrobrachium nipponense* through comparative transcriptomic analysis

Hui Qiao[1], Hongtuo Fu[1]*, Yiwei Xiong[1], Sufei Jiang[1], Wenyi zhang[1], Shengming Sun[1], Shubo Jin[1], Yongsheng Gong[1], Yabing Wang[2], Dongyan Shan[3], Fei Li[2], Yan Wu[1]

1 Key Laboratory of Freshwater Fisheries and Germplasm Resources Utilization, Ministry of Agriculture, Freshwater Fisheries Research Center, Chinese Academy of Fishery Sciences, Wuxi 214081, China

2 Wuxi Fishery College Nanjing Agricultural University, Wuxi 214081, China

3 College of Fisheries and Life Sciences, Shanghai Ocean University, Shanghai 201306, China.

*Corresponding author: Hongtuo Fu

Freshwater Fisheries Research Center, Chinese Academy of Fishery Sciences

9 Shanshui East Road

Wuxi 214081, Jiangsu Province, P. R. China

E-mail address: fuht@ffrc.cn

Tel: +86 510 85558835

Fax: +86 510 85553304

Table S1 the specific primers used to in qRT-PCR

Primer name	Sequence（5′→3′）
opsin	F: GTAATACACTGCCATCCACGAAC
	R: CAATAGCGAGGAGGCCCATAAC
early cuticle protein 6	F: GAAGTGTGTATTGCTTTCCGTCTT
	R: CTCTGAGTAGACGATGCCGTTG
calcification associated soluble matrix protein 2	F: TTGGTTCTTACAGCTACCCACAG
	R: TGTTTCCTTCACGAGCCTTACG
Tubulin beta-1 chain	F: TACTACAATGAAGGCAACCAGGG
	R: GTATAGTGACCTTTGGCCCAGTT
myosin essential light chain	F: CATCTTCGACCAGAAGGGTGATG
	R: GAAATCGTCAGCAGTGTCAACAG
neuroparsin	F: AATTCCTTCCGCTCCTTTGTCAT
	R: TCAAGATTTCCGCACCATCCAA
crustacean cardioactive peptide	F: GTTGCTAAAAGGGACATTGGTGG
	R: TTGTTGTGAGGATGCTGCTAACT
tachykinin	F: ATGTATAGTGTTGATGGGCGTGAT
	R: ATTGGGTAGTCGTCCTCCTGTA
pigment-dispersing hormone 3	F: TTGCTACGCAGTGGCCCAGGAA
	R: TGCTTCAGTCATGACCCTGGGAA
neuropeptide F II	F: CGCAGATTCAGGCTTTGGAAAA

R: GCCAAAGTCTCCATCAGTCTCTC

Table S2 Summary of functional annotation of *M. nipponense*

Annotated databases	Unigene	⩾300 bp	⩾1000 bp
COG	9,646	5,428	4,218
GO	1,3295	8,696	4,599
KEGG	9,243	5,176	4,067
Swissprot	22,381	13,116	9,265
NR	33,012	21,208	11,804
All	34,183	22,282	11,901

Table S3 SSR analysis of transcriptome data of *M. nipponense*

Searching item	Numbers
Total number of sequences examined	17844
Total size of examined sequences (bp)	38700240
Total number of identified SSRs	14889
Number of SSR containing sequences	8745
Number of sequences containing more than 1 SSR	3667

Number of SSRs present in compound formation	1416
Mono-nucleotide	7953
Di-nucleotide	3382

Table S4 SNP analysis of transcriptome data of *M. nipponense*

Sample ID	Homo SNP	Hete SNP	All SNP
BSE-1	106,965	147,703	254,668
BSE-2	109,725	129,399	239,124
BSE-3	110,393	125,829	236,222
BSB-1	140,902	72,507	213,409
BSB-2	135,891	69,020	204,911
BSB-3	135,042	67,282	202,324
NBSE-1	135,232	57,507	192,739
NBSE-2	130,875	52,094	182,969
NBSE-3	129,451	50,852	180,303
NBSB-1	148,332	53,786	202,118
NBSB-2	142,845	49,641	192,486

NBSB-3	141,437	48,373	189,810
Total	1,567,090	923,993	2,491,083

Table S5 Summary statistics of differentially expressed genes

DEG Set	DEG Number	up-regulated	down-regulated
NBSE vs BSE	3,271	2,136	1,135
NBSB vs BSB	2,014	1,318	696

Table S6 Functional annotation analyses of DEGs

**DEG Set**	**Total**	**GO**	**KEGG**	**COG**	**Swiss-Prot**	**NR**
NBSE vs BSE	2,288	1,076	552	523	1,796	2,254
NBSB vs BSB	1,410	650	375	417	1,075	1,377

Table S7 highly significantly enriched GO terms of DEGs (top 10 terms)

**No.**	**GO.ID**	**GO Name**	**Annotated**	**Significant**	**Expected**	**KS**
1	GO:0009881	photoreceptor activity	66	52	6.28	2.60E-29
2	GO:0042302	structural constituent of cuticle	122	51	11.6	6.00E-14
3	GO:0004930	G-protein coupled receptor activity	177	58	16.83	1.10E-11
4	GO:0005200	structural constituent of cytoskeleton	153	40	14.55	2.20E-08

5	GO:0008601	protein phosphatase type 2A regulator activity	20	0	1.9	1.10E-06
6	GO:0005506	iron ion binding	130	18	12.36	4.80E-06
7	GO:0005509	calcium ion binding	229	37	21.78	7.90E-06
8	GO:0030414	peptidase inhibitor activity	50	21	4.76	8.20E-06
9	GO:0031409	pigment binding	13	9	1.24	3.20E-05
10	GO:0005525	GTP binding	359	54	34.14	4.70E-05

Notes: Annotated：The number of all genes annotated in this GO term; Significant: The number of DEGs annotated in this GO term; Expected：Expected value of DEGs annotated in this GO term;；KS：The *p* - value of Kolmogorov - Smirnov test.

Table S8 Neuropeptides discovery by transcriptome mining and differentially expressed analyses

Gene ID	Neuropeptides	NBSE/BSE			NSB/BSB			Species	E-value	Ident
		+/-	FDR	FC	+/-	FDR	FC			
**Prawn40668_c0_g1**	red pigment concentrating hormone(RPCH)	/	/		/	/		*Macrobrachium rosenbergii*	1.92e-57	98.97
**Prawn43145_c0_g1**	crustacean hyperglycemic hormone(CHH)	/	/		/	/		*Macrobrachium rosenbergii*	1.20e-85	97.01
**Prawn43868_c0_g1**	molt inhibiting hormone(MIH)	/	/		/	/		*Macrobrachium nipponense*	1.7e-80	99.16
**Prawn42069_c0_g1**	gonad inhibiting hormone(GIH)	/	/		/	/		*Macrobrachium nipponense*	1.07e-74	100.00
**Prawn36457_c0_g1**	allatostatin-B- I(AST-B-I)	/	/		/	/		*Pandalopsis japonica*	3.87e-107	83.63
**Prawn28326_c0_g1**	allatostatin-B- II I(AST-B- II)	/	/		/	/		*Pandalopsis japonica*	3.83e-55	73.33
**Prawn51639_c0_g1**	allatostatin-C(AST-C)	/	/		/	/		*Pandalopsis japonica*	1.58e-59	74.83
**Prawn24321_c0_g4**	neuropeptide F I(NPF-I)	/	/		/	/		*Litopenaeus vannamei*	7.55e-09	83.33
**Prawn24321_c0_g14**	neuropeptide F II I(NPF-II)	-	2.35E-04	3.17	/	/		*Litopenaeus vannamei*	2.98e-63	81.60
**Prawn42177_c0_g1**	short neuropeptide F (sNPF)	/	/		/	/		*Aedes aegypti*	6.21e-07	30.72
**Prawn29703_c0_g1**	sulfakinin	/	/		/	/		*Homarus americanus*	3.38e-39	64.60
**Prawn51952_c2_g1**	neuroparsin (NP)	+	3.50E-16	0.07	-	3.09E-05	15.62	*Jasus lalandii*	6.85e-30	51.00
**Prawn40940_c0_g3**	orcokinin II	-	5.02E-04	3.34	/	/		*Homarus americanus*	8.33e-66	81.45
**Prawn38249_c0_g1**	crustacean cardioactive peptide (CCAP)	-	4.83E-05	3.61	/	/		*Homarus gammarus*	8.98e-48	65.44
**Prawn49450_c0_g1**	eclosion hormone 2 (EH2)	/	/		/	/		*Nilaparvata lugens*	5.90e-18	50.70
**Prawn52274_c3_g1**	pigment dispersing hormone I (PDH-I)	/	/		/	/		*Marsupenaeus japonicus*	8.26e-07	64.29

Gene ID	Neuropeptides	NBSE/BSE			NSB/BSB			Species	E-value	Ident
		+/-	FDR	FC	+/-	FDR	FC			
**Prawn47964_c0_g6**	pigment-dispersing hormone 3(PDH3)	-	1.53E-03	3.57	/	/		*Marsupenaeus japonicus*	4.03e-17	64.18
**Prawn52949_c0_g1**	tachykinin(TK)	-	2.08E-03	4.00	/	/		*Homarus americanus*	1.74e-06	49.12
**Prawn52168_c1_g1**	Calcitonin-like diuretic hormone	/	/		/	/		*Homarus americanus*	9.65e-62	72.73
**Prawn37512_c0_g1**	corazonin preprohormone	/	/		/	/		*Daphnia pulex*	2.76e-06	79.31
**Prawn40208_c0_g2**	crustacean female sex hormonoe	/	/		/	/		*Carcinus maenas*	1.03e-30	37.66
**Prawn50466_c0_g1**	FLRFamide precursor protein B	/	/		/	/		*Procambarus clarkii*	1.34e-36	33.84

Note: +means significant up-regulated expression; - means significant down-regulated expression. FC means fold change of expression level of the comparative group.

Figure S1 Species distribution of BLASTx hits

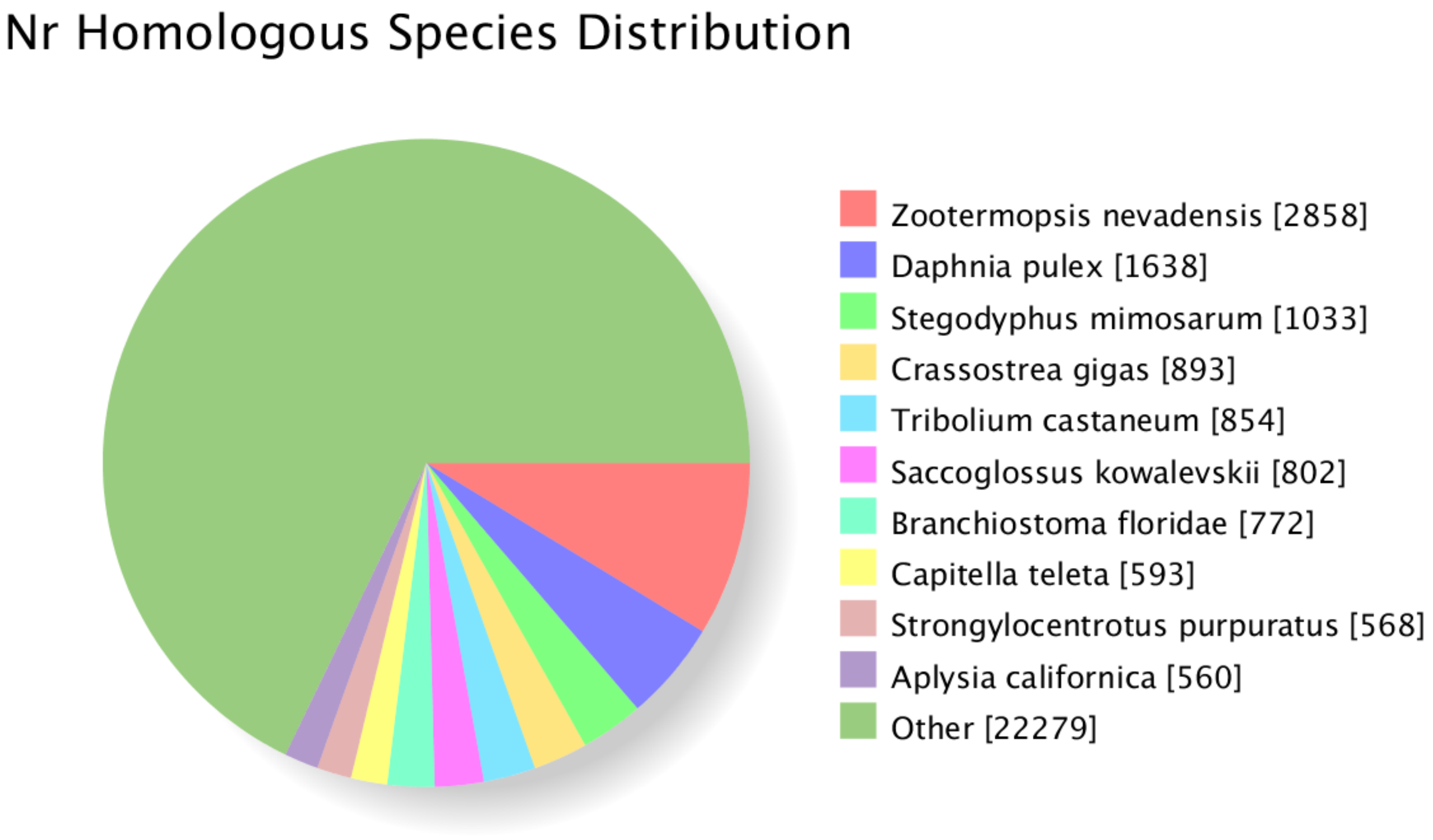

Figure S2 Gene Ontology (GO) categorization for assembled unigenes

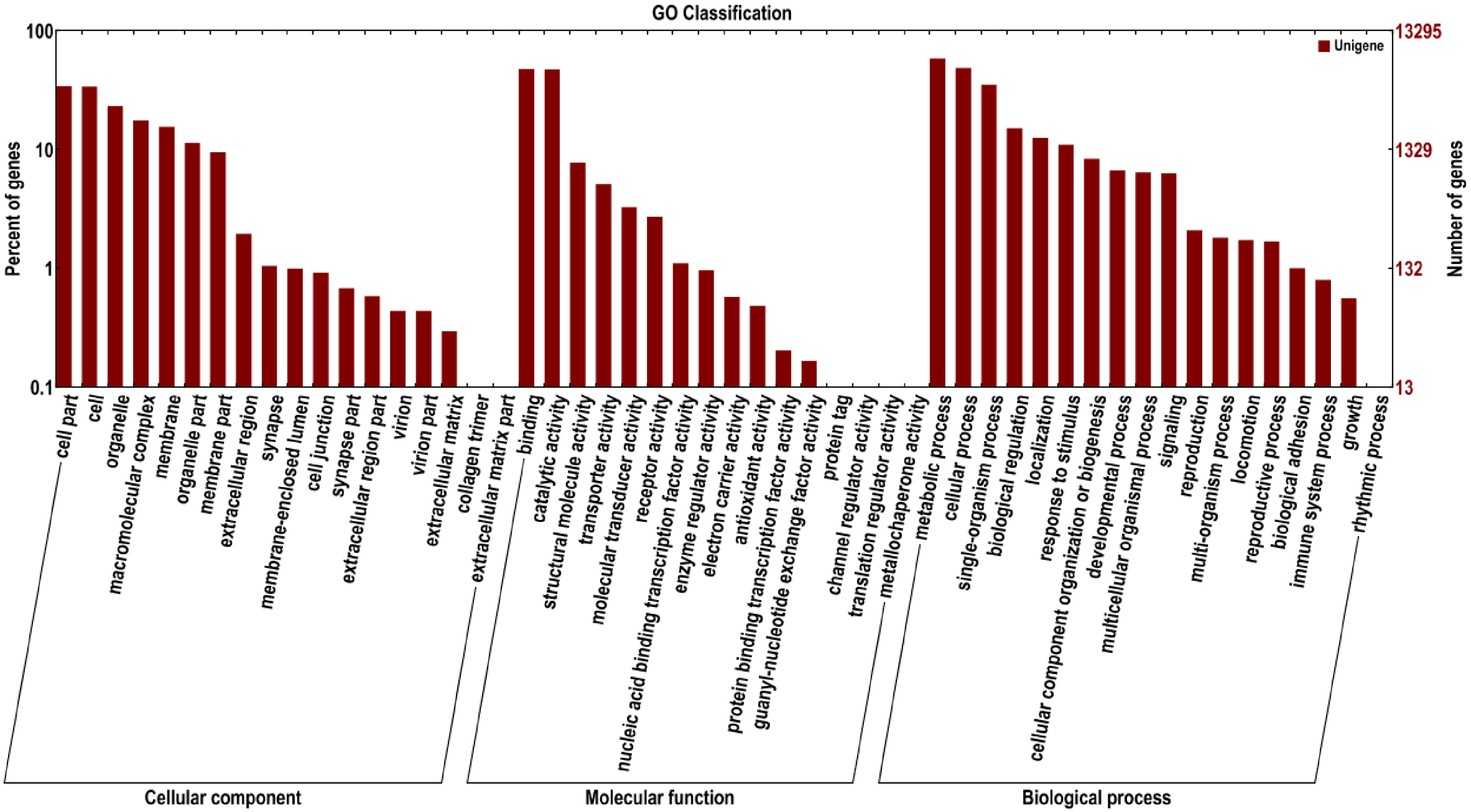

Figure S3 Clusters of Orthologous Groups (COG) functional classification of *M. nipponense*

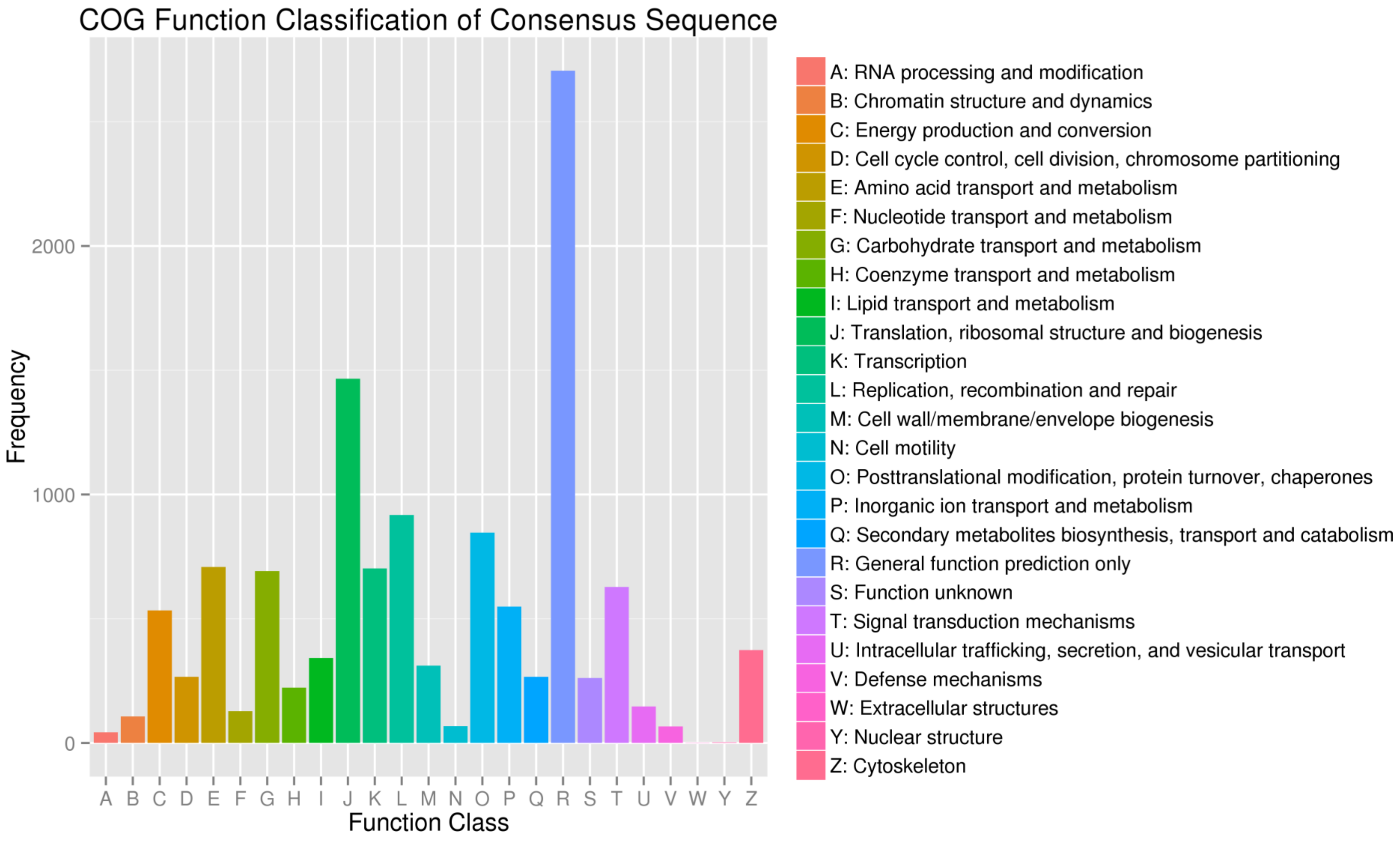

Figure S4. Comparison between RT-qPCR data and RNA-Seq.

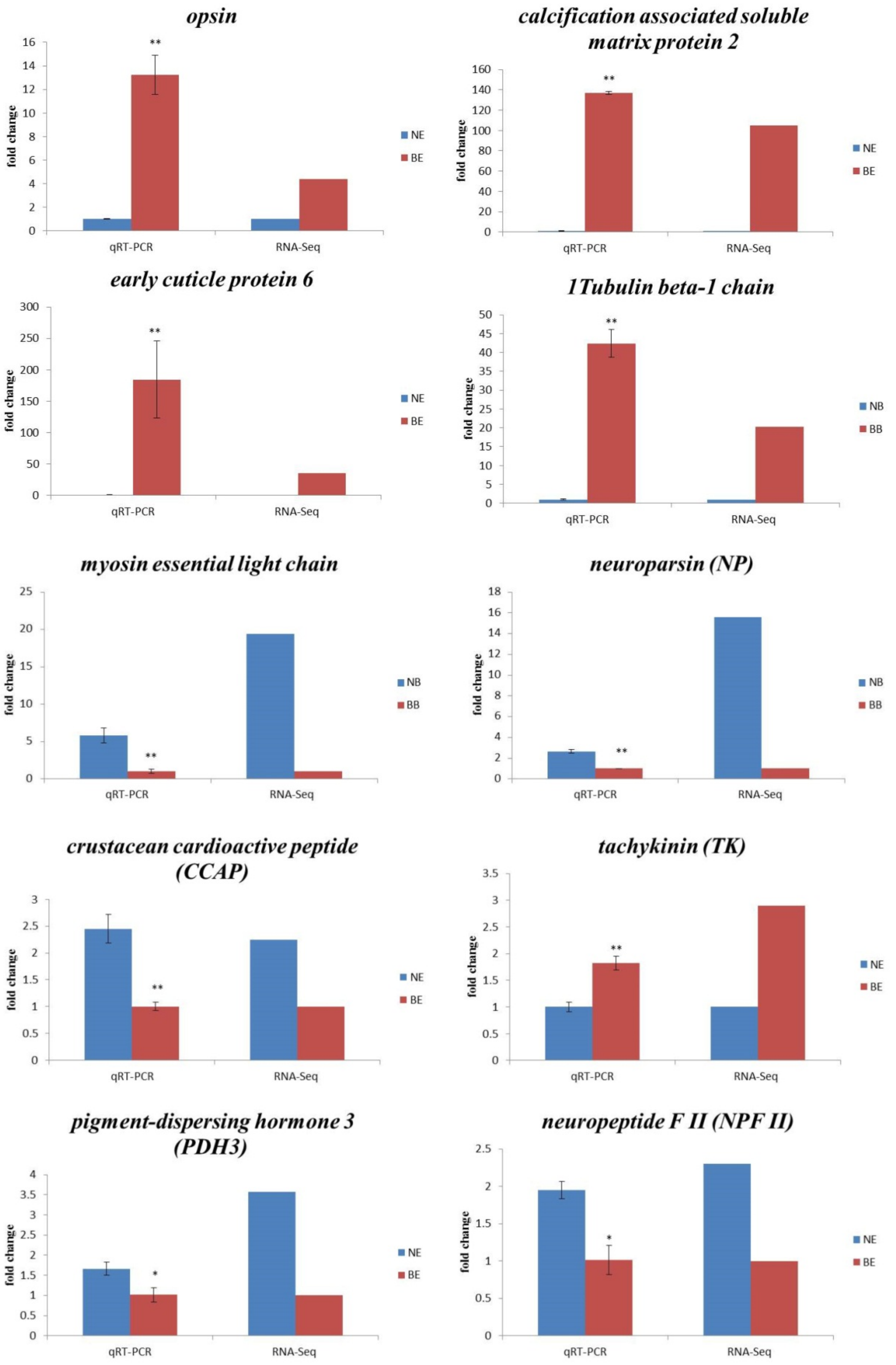

Supplement: Supplementary file 1 — Supplementary Info [file 41598_2017_10439_MOESM1_ESM.pdf]
